# Receptor-Associated Protein Blocks Internalization and Cytotoxicity of Myeloma Light Chain in Cultured Human Proximal Tubular Cells

**DOI:** 10.1371/journal.pone.0070276

**Published:** 2013-07-23

**Authors:** Sule Sengul, Sehsuvar Erturk, Altaf M. Khan, Vecihi Batuman

**Affiliations:** 1 Department of Nephrology, Ankara University School of Medicine, Ankara, Turkey; 2 Division of Nephrology, Tulane University School of Medicine, New Orleans, Louisiana, United States of America; 3 SLVHCS, VA Medical Center, New Orleans, Louisiana, United States of America; Aarhus University, Denmark

## Abstract

**Background:**

Free light chains (LCs) are among the many ligands that bind to cubilin/megalin for endocytosis via the clathrin-dependent endosomal/lysosomal pathway. Receptor associated protein (RAP), is a 39 kDA high-affinity, chaperone-like ligand for megalin that assists in the proper folding and functioning of megalin/cubilin. Although RAP is known to inhibit ligand binding to megalin/cubilin, its effect on LC endocytosis has not been shown directly.

**Methods and Principal Findings:**

We investigated whether RAP can block the endocytosis of LC in cultured human proximal tubule cells and whether this can prevent LC cytotoxicity. Immunofluorescence microscopy and flow cytometry showed that fluorescently labeled LC endocytosis was markedly inhibited in HK-2 cells pretreated with human RAP. The effect of RAP was dose-dependent, and was predominantly on endocytosis as it had no effect on the small acid-washable fraction of LC bound to cell membrane. RAP significantly inhibited LC induced cytokine production and phosphorylation of ERK1/2 and p38 MAPK. Prolonged exposure to LC for 48 h resulted in epithelial-to-mesenchymal transformation in HK-2 cells as evidenced by marked reduction in the expression of the epithelial cell marker E-cadherin, and increased the expression of the mesenchymal marker α-SMA, which was also prevented by RAP in the endocytosis medium.

**Conclusions:**

RAP inhibited LC endocytosis by ∼88% and ameliorated LC-induced cytokine responses and EMT in human PTCs. The results not only provide additional evidence that LCs endocytosis occurs via the megalin/cubilin endocytic receptor system, but also show that blocking LC endocytosis by RAP can protect proximal tubule cells from LC cytotoxicity.

## Introduction

Myeloma light chains (LCs) are low-molecular weight proteins, relatively freely filtered in the glomeruli, endocytosed by a receptor-mediated process in the proximal tubule cells (PTCs) through the tandem endocytic receptors megalin/cubilin and targeted to degradative sites [1]–[4]. We have observed that excessive endocytosis of LC leads to cellular protein overloading and triggering a sequence of inflammatory processes through the activation of intracellular signaling pathways involving mitogen activated protein kinases (MAPKs), and transcription factors, nuclear factor kappa B (NF-κB) and activator protein-1 (AP-1) complexes and production of inflammatory cytokines [5]–[8]. Endocytosis is a prerequisite for the toxic effects of LCs on PTC that include these inflammatory events as well as direct cytotoxicity, such as, transport abnormalities, cell injury (apoptosis and necrosis) and epithelial-mesenchymal transition [7], [9]–[16]. These inflammatory and cytotoxic phenomena provide the basis of both chronic tubulointerstitial disease and acute renal injury associated with myeloma [10], [16].

Receptor-associated protein (RAP) serves as a molecular chaperone/escort protein for megalin/cubilin to ensure safe transport of receptor-protein complex to degradative sites, and is necessary for the proper functioning of this receptor system [17]. RAP is a competitive inhibitor of other ligands of megalin/cubilin complex [18]–[20]. It is known to inhibit the binding of many but not all ligands to megalin [21], [22]. Since LC is well characterized as a megalin ligand, RAP would be expected to antagonize LC binding, but its role in LC endocytosis has not been evaluated directly. Furthermore, previous observations have shown that blocking the endocytosis of LCs by a variety of maneuvers including silencing megalin and cubilin expression in PTC protects kidney proximal tubule cells from LC cytotoxicity [7], [12], [13], [23]. Whether RAP inhibition of LC internalization could also protect cells from cytotoxic effects of LC has not been studied. We hypothesized that RAP would inhibit the endocytosis of LC like most other ligands of megalin, and this would protect the cells from LC cytotoxicity. We therefore studied the role of RAP in the endocytosis and cytotoxicity of myeloma LC in cultured human PTCs.

## Methods

### Isolation and Purification of Myeloma LCs

LCs were collected anonymously from myeloma patients with modest renal insufficiency without albuminuria and no evidence of glomerular involvement. Thus the LCs studied here are considered tubulopathic. Kidney biopsies were not performed in the patients. The LCs used in the present study were previously shown to undergo receptor-mediated endocytosis by PTCs, bind to cubilin and megalin, and induce cytokines through phosphorylation of MAPKs and activation of NF-κB [1]–[4], [6], [7], [14], [23]. LCs were isolated and purified by a slight modification of the method previously reported from our laboratory [2], [7], [14]. Briefly, LCs were precipitated from urine with ammonium sulfate (55 to 90% saturation–determined empirically), extensively dialyzed against distilled water and lyophilized. The precipitated LCs were purified by dissolving the lyophilized desalted crude protein in potassium phosphate-based buffer at pH 6.0 (100 ml 0.1 M KH2PO4+11.2 mls of 0.1 M NaOH), followed by chromatography on carboxymethyl-Sephadex (C-50; Pharmacia, Piscataway, NJ, USA). Under these conditions, the LCs were bound to the column, whereas the contaminants were not. Bound LC was eluted with 0.6 mol/L of NaCl, redialyzed against distilled water, and lyophilized. The purity of LCs was confirmed by sodium dodecyl sulfate (SDS) gel electrophoresis and the immunologic identity reported from the clinical laboratory was confirmed by Western blotting using goat antihuman κ and λ antibodies.

The LC preparations were tested for endotoxin using the chromogenic Limulus amebocyte lysate (LAL) test (Charles River Labs, Charleston, SC, USA). Endotoxin has previously been shown to induce MCP-1 gene expression through activation of nuclear factor-κB (NF-κB) in rat tubular epithelial cells at 1 to 10 µg/mL concentrations corresponding to approximately 3000 to 30,000 EU according to the manufacturer’s specifications. We found that all LCs used in our laboratory were essentially endotoxin free (0.1 to 3.7 EU/mg LC protein). The LCs used in these experiments did not contain detectable quantities of interleukin (IL)-1, IL-6, IL-8, monocyte chemoattractant protein (MCP)-1 or tumor necrosis factor (TNF)-α, indicating that cytokine contamination does not occur during purification.

### Cell Cultures

HK-2 human renal PTCs were purchased from the American Type Culture Collection (Rockville, MD) and were maintained in Gibco Keratinocyte-Serum-Free Medium supplemented with 5 ng/ml recombinant EGF and 0.05 mg/ml bovine pituitary extract (Invitrogen, Carlsbad, CA). These cells are not passaged forward beyond about 25–30 passages and were routinely cultured at 37°C in a humidified atmosphere of 95% air–5% CO_2_ and nourished at intervals of 3–4 days. At desired level of confluence (∼80%) as the experimental protocol requires, the culture medium is aspirated, the cultures are rinsed with phosphate-buffered saline (PBS), the cells are removed by trypsin/ethylene-diamine tetraacetic acid digestion and re-seeded into 6-well or 12-well plates for experiments. These cells are known to express both cubilin and megalin, and endocytose LCs through cubilin/megalin binding [1], [2], [23].

### Immunofluorescence Microscopy and Flow Cytometry Analysis of LC Endocytosis and the Effect of RAP

The effect of human RAP (Innovative Research, Novi, MI) pre-treatment (1 μM, 1 h) on the uptake of fluorescein isothiocyanate (FITC) labeled human myeloma LC (25 μM) by human PTCs was tested by immunofluorescence microscopy and flow cytometry. Human κ-LC was FITC-conjugated using a FluoroTag FITC Conjugation Kit (Sigma, St. Louis, MO) as described previously [23]. The confluent HK-2 cells in 6-well plates were treated with 1 µM human RAP for 1 h at 37°C before exposure to 25 µM FITC-labeled κ-LC.

**Figure 1:**
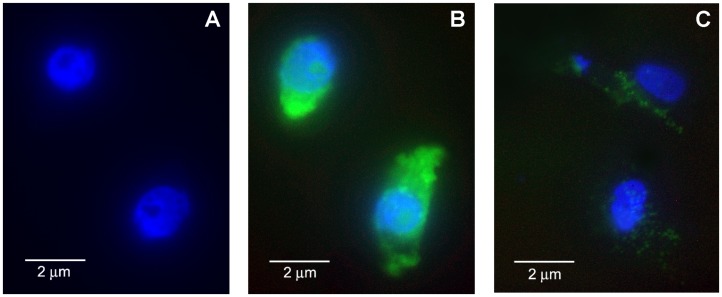
Endocytosis of FITC-κLC in cultured human proximal tubule epithelial cells (a representative field with two cells is shown). HK-2 cells were grown on a glass chamber in 8-well culture slide for 2 days and then observed by fluorescence microscopy. **A.** FITC without LC in control cells counterstained with DAPI for 30 min. **B:** after FITC-LC (25 µg/ml) uptake by HK-2 cells grown on slide chamber for 45 min at 37°C after acid wash to strip membrane bound fluorescence and counterstained with DAPI shows distribution of FITC-LC in the cytosol (green fluorescence). **C:** Pretreatment with 1 uM receptor-associated protein (RAP) markedly inhibits FITC-LC fluorescence in HK-2 cells.

Cells exposed to FITC alone without LC, and to FITC-LC for 45 min at 37°C before and after 1 µM RAP treatment were grown in a glass chamber, counterstained with 4′,6-diamidino-2-phenylindole (DAPI) for immunofluorescence microscopy, and photographed in a fluorescence microscope (Figure 1). To visually confirm internalization of LC cells were photographed after acid washing to strip any cell-surface bound LC using an acid solution (50 mM glycine and 100 mM NaCl, pH 5.0) as previously reported from our laboratory [3].

**Figure 2:**
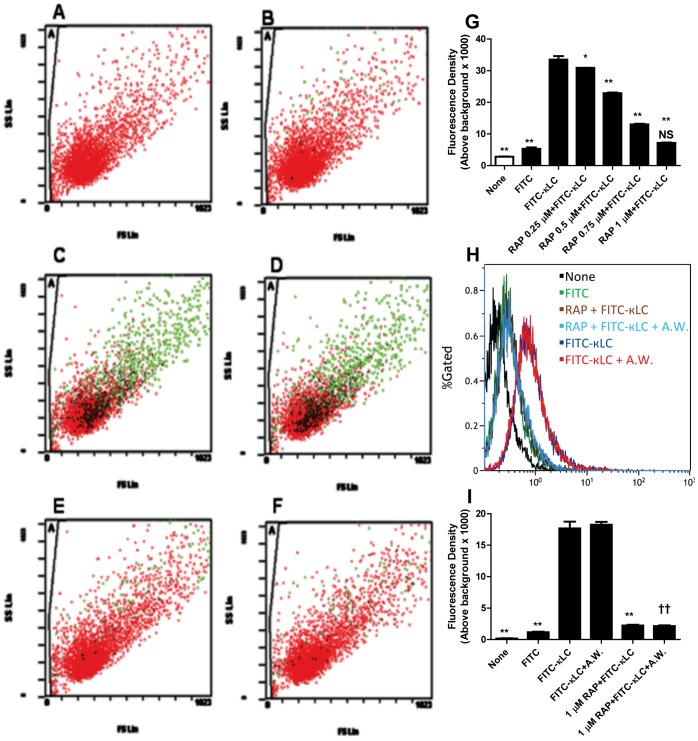
Flow cytometry (A – F) showing the effect of RAP treatment on the association of fluorescently labeled human myeloma light chain (LC) by human renal proximal tubule cells. Cells with FITC association are represented in green color. (**A** = cells without LC, **B** = cells with FITC alone, **C** = cells with 25 µM FITC-κLC, **D** = cells with 25 µM FITC-κLC+acid wash, **E** = cells after 1 µM RAP and with 25 µM FITC-κLC, **F** = cells after 1 µM RAP +25 µM FITC-κLC+acid wash). Quantitative presentation of flow cytometric events showing the inhibitory effect of RAP on the association of 25 µM FITC-κLC in HK2 cells is dose-dependent **G**). *p<0.05, **<0.01 compared to 25 µM FITC-κLC, N.S. = Not significant compared to FITC alone. Histograms (**H**) represent the distributions of events for FITC-LC associated cells. Acid-wash had no discernible effect on the magnitude of FITC-LC association with the cells, suggesting that membrane association is negligible. The percent magnitude of fluorescence above background is shown in the bar graph (**I**): Control non-FITC conjugated LC = 0.18±0.03%; FITC alone = 1.16±0.10; 25 uM FITC- conjugated κ-LC = 17.59±1.06%; 25 uM κ-LC+A.W = 18.25±0.46%; 1 uM RAP +25 uM FITC-κ-LC = 2.24±0.11%; 1 uM RAP +25 uM FITC- κ-LC+A.W. = 2.14±0.11%. RAP, 1 µM, reduced the association of total FITC-LC with cells by 87.3% compared to cells exposed to FITC-κ-LC (down to 2.24±0.11 from 17.59±1.06%), and reduced acid non-washable fraction (the fraction endocytosed by cells) by 88.3%, (reduced from 18.25±0.46 to 2.14±0.11%; Mean ± SE of three experiments, **p<0.01 compared to 25 µM FITC-κ-LC, †† p<0.01 compared to 25 µM FITC-κ-LC+A.W.; A.W. = Acid Wash).

For flow cytometry, the cells were washed several times with PBS, trypsinized, and re-suspended in PBS. Fluorescence in cells exposed to FITC-LC for up to 45**min at 37°C was detected by using a Beckman Coulter FC500 Cytomics with CXP software. The positive control was FITC-labeled κ-LC without RAP treatment; the negative control was unlabeled κ-LC. To determine the percentage of endocytosed LC, cells were washed by an acid solution to remove any cell surface bound FITC-LC (Figure 2). Dose response studies showed a dose-dependent inhibition of endocytosis of LCs by HK2 cells with maximum inhibitory effect of RAP seen at 1 µM concentration in the medium (Figure 2G). All experiments on its effects were conducted at this concentration of RAP.

### The Effect of RAP Pretreatment on LC Cytotoxicity

To test the effect of RAP pre-treatment on cytotoxic responses, the cells were pre-treated with RAP, 1 µM, for at least 1 h prior to the addition of LCs and subsequently co-treated with myeloma LC (25 µM) for 4–48 h. LC concentration (25 µM) in this study was chosen to approximate the concentration range reached in the glomerular ultrafiltrate in patients with modest proteinuria and was previously demonstrated to elicit cytokine responses in human PTC *in vitro* [7].

To test the effect of RAP on cytokine release, cultured human PTCs were preincubated with recombinant RAP, 1 µM, for 1 h and exposed to 25 µM κ-LC for 4 h in the continuous presence of RAP. At the end of exposure period, supernatants were harvested and stored at −70°C for cytokine measurements. Cytokines, interleukin (IL)-6 and 8, were measured by enzyme-linked immunosorbent assay (ELISA) in the supernatants of PTCs [7]. This concentration of RAP had no effect on basal cytokine production.

For blotting experiments, after pretreatment with RAP for 1 h, confluent monolayers of serum-deprived PTCs were treated with LC for up to 48 h in six-well plates. At the end of exposure, cell lysates were blotted for MAPKs, E-cadherin and α-SMA. Cell viability was tested by CellTiter 96 Aqueous One solution Cell Proliferation Assay (Promega, Madison, MI).

### Measurement of IL-6 and IL-8 Levels by ELISA

IL-6 and IL-8 were measured in the supernatants using commercial human ELISA kits (Quantikine; R&D Systems) according to the manufacturer’s protocol. The sensitivity of the ELISA is 0.7 pg/ml for IL-6 and 10 pg/ml for IL-8 assays. Cytokine concentrations in the unknown samples were determined by comparison with a standard curve developed with known amounts of recombinant human cytokines provided with the kits. Experiments were conducted in triplicate using 96-well microplates, and results were read in a microplate reader. Cells were trypsinized and counted and expressed as ng of cytokine per 10^6^ cells.

### Preparation of Whole Cell Lysates

Cells planted onto 35-mm sterile tissue culture dishes (Corning Glass Works, Corning, NY) or six-well tissue culture plates were grown at 37°C in serum-free medium in an incubator for 24 h. After each experiment, medium was removed and cells were washed with PBS. The following steps were done on ice: 200 µl of RIPA buffer consisting of 50 mM Tris · HCl (pH 7.4), 1% NP-40 (IGEPAL-CA630), 0.25% Na-deoxycholate, 150 mM NaCl, 1 mM EDTA (EGTA) (pH 8.0), protease inhibitors (aprotinin, leupeptin, and pepstatin, 1 µg/ml each, and freshly added), 1 mM Na_3_VO_4_, and 1 mM PMSF (added immediately before use) were added to the dishes. After 10 min of incubation, dishes were scraped with a cell scraper and then lysates were transferred to 1.5-ml microcentrifuge tubes using syringes fitted with 21-gauge needles. Lysates were passed through a 21-gauge needle to shear DNA and centrifuged at 13,000 *g* for 10 min at 4°C. Finally, supernatants were harvested as whole cell lysates and used in immunoblotting studies. Protein concentrations were determined in whole cell lysates prepared from PTCs by using Pierce BCA Protein Assay (Rockford, IL).

### Western Blot Analysis for MAPKs, E-cadherin and α-SMA

Phosphorylation of MAPKs, and the expression of E-cadherin and α-SMA were evaluated in the whole cell lysates by Western blotting. Equal amounts of proteins were separated by NuPAGE Bis-Tris gel by using precast gels (10% acrylamide) and a minigel apparatus (Novex). Electrophoresis was performed according to manufacturer’s recommendations. Separated proteins were electrophoretically transferred to nitrocellulose membranes for 1 h at 30 V by using a semi-dry blotting apparatus (Novex). Proteins were probed with polyclonal phospho-specific antibodies against ERK 1/2 and p38 (Cell Signaling, Beverly, MA) at 1∶5,000 and 1∶5,000 dilutions, respectively by using a Western Breeze Chemiluminescent Kit according to the manufacturer’s protocol (Novex). Integrity of phospho-specific antibodies against MAPKs was tested using sorbitol and nerve growth factor-treated PC12 cell extracts (positive controls) provided by the antibody supplier (Promega, Madison, WI). We determined total MAPKs using primary antibodies (1∶1,000 dilution, Cell Signaling, Beverly, MA) to confirm equal protein loading in the gels for each experiment. Representative blots from one of at least three separate experiments were selected for illustration.

Protein extracts (20 µg) were also separated by 12.5% SDS-PAGE, electrophoretically transferred onto a nitrocellulose membrane, and then incubated with appropriate dilutions of the primary antibody (1∶500 monoclonal anti-E-cadherin [G-10, Santa Cruz Biotechnology, Santa Cruz, CA], 4.2 µg/ml monoclonal anti-α-smooth muscle actin (α-SMA) [clone 1A4, Sigma] or 0.9 µg/ml monoclonal anti-actin [clone AC-40, Sigma]). After four washes in TBST buffer, the membrane was incubated with horseradish peroxidase-conjugated sheep anti-mouse immunoglobulin (GE Healthcare, Piscataway, NJ), and the antibody complexes were visualized by the Amersham ECL detection system (GE Healthcare) as directed by the manufacturer.

### Statistical Analysis

Results were expressed as means ± SE. Multiple comparisons were made by ANOVA using Newman-Keuls Multiple Comparison Test. Statistical analyses, curve fitting, and calculations were done using GraphPad Prism, version 4 for Macintosh (2004), GraphPad Software, San Diego, CA). Statistical significance was defined as *P*<0.05.

## Results

### Effect of RAP Pretreatment on LC Endocytosis

Inhibition of LC endocytosis by RAP is demonstrated by immunofluorescence microscopy (Figure 1), and flow cytometry (Figure 2). Both experiments showed that fluorescently labeled human myeloma LC endocytosis is markedly inhibited in HK-2 cells pretreated with 1 μM human RAP. The inhibitory effect of RAP on LC endocytosis was dose-dependent and maximal inhibition was observed at 1 µM (Figure 2G). Cells were washed with an acid solution after exposure to FITC-LC to strip any surface-bound LC, and photographed and analyzed by flow-cytometry before and after acid-wash. Flow cytometric analysis of the cells permitted quantitative assessment and revealed that nearly all of the cell-associated FITC-LC remained in the cells after the surface bound LC was removed by acid washing of the cells. RAP, 1 µM, markedly inhibited (by ∼88%) the acid non-washable FITC- κ LC association, i.e. endocytosis, by the cells (Figure 2). These results are remarkably similar to our previous observations with radiolabeled LCs, which showed that ∼80% proximal tubule cell associated LC is acid non-washable [3].

### Effect of RAP Pretreatment on LC-induced IL-6 and IL-8 Production

**Figure 3:**
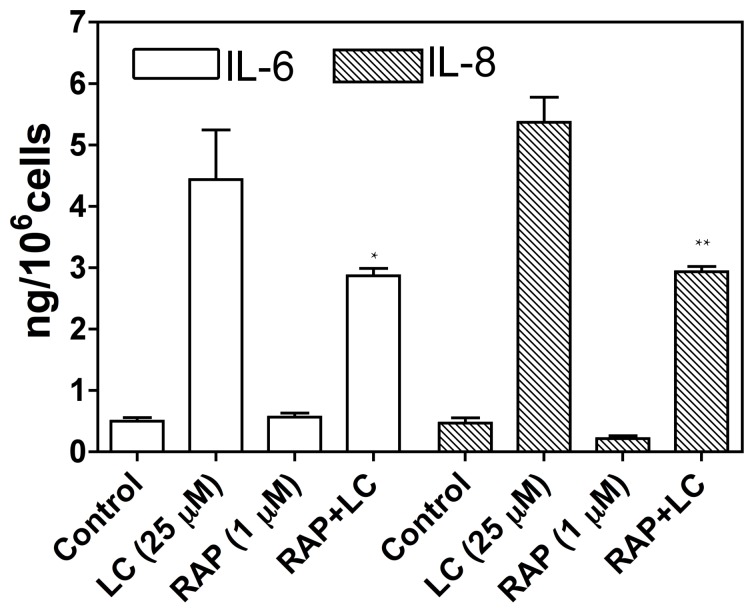
Effect RAP on myeloma light chain (LC)- stimulated cytokine production. Cells were pre-incubated for 1-h with or without 1 µM RAP and then exposed to 25 µM myeloma LC for a further 4-h. Interleukins 6 and 8 were then assayed in the cell supernatants. LC-stimulated the production of both IL-6 and IL-8, and both were significantly inhibited by the presence of RAP, 1 µM, in cell culture medium (p<0.05). Bars represent the mean ± SE for each group (n = 3, *p<0.05 vs LC, **p<0.01 vs LC, one-way ANOVA).

To confirm the contribution of receptor-mediated endocytosis of myeloma LC to cytotoxicity in human PTCs, we tested the effect of RAP interference on ligand binding to megalin/cubilin complex. After 1-h pretreatment with 1 µM RAP, myeloma LC-stimulated production of cytokines IL-6 and 8 was significantly inhibited by co-incubation with RAP (Figure 3). Thus, inhibition of LC endocytosis through RAP competition for megalin/cubilin binding significantly reduced LC toxicity. RAP alone had no significant effect on cytokine production in cultured human PTCs (Figure 3).

### Effect of RAP Pretreatment on LC-induced MAPKs Activation and EMT

**Figure 4:**
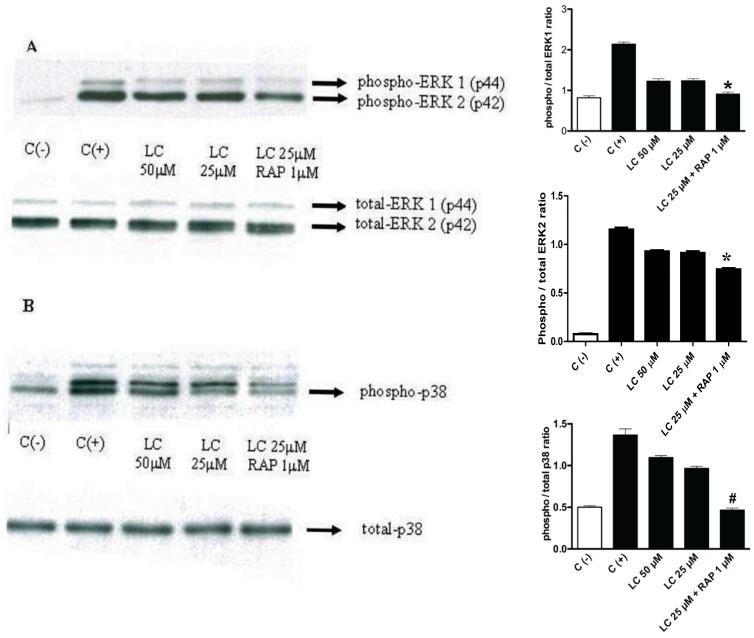
Effect of LCs on ERK1/2 and p38 MAPK (immunoblots on the left side of the panel with corresponding bar graphs of densitometric measurements next to each immunoblot panel. Panel **A** shows inhibition of light chain (LC)-stimulated phosphorylation of ERK ½, and panel **B**, inhibition of phosphorylation of p38 MAPK by RAP pretreatment. After 1-h pretreatment with 1 µM RAP human proximal tubule cells were exposed to LC for 4 h (representative blots from 3 different experiments are shown; “C (−)” = negative control; “C (+)” = positive control; bars represent mean ± SE of three densitometric readings). ***A*** *:* Effect of RAP pretreatment on LC-induced activation of ERK1/2. Cells were pretreated with RAP for 1 h and then treated with LC in the continued presence of RAP for 4 h. ***Top***: RAP inhibited LC-induced activation of ERK1/2 (LC 25 µM+RAP 1 µM vs. positive control, C (+), *P<0.001; LC 25 µM+RAP 1 µM vs. LC 25 µM alone, P<0.05; LC 25 µM+RAP 1 µM vs. LC 50 µM alone, P<0.01; LC 25 µM+RAP 1 µM compared to negative control, C (−), P>0.05, Newman-Keuls Multiple Comparison Test). ***Bottom***: Total ERK was not affected. ***B*** *:* Effect of RAP pretreatment on LC-induced activation of p38 MAPK. *Top:* LC-induced activation of p38 was suppressed by RAP (LC 25 µM+RAP 1 µM vs. C (+), P<0.001; LC 25 µM+RAP 1 µM vs. LC 50 µM alone, # P<0.001. LC 25 µM+RAP 1 µM vs. LC 25 µM alone, P<0.001; LC 25 µM+RAP 1 µM vs. C (−), P>0.05, Newman-Keuls Multiple Comparison Test). ***Bottom***: Total p38 was not affected (representative blots from 3 different experiments are shown).

To test the effect of RAP on activation of MAPKs after 1-h pretreatment with RAP (1 µM), human PTCs were exposed to myeloma LC (25 µM) for up to 4 h. Myeloma LC-stimulated phosphorylation of ERK1/2 and p38 MAPK was inhibited by co-incubation with RAP (Figure 4A & 4B). Blots for total MAPKs remained constant throughout the duration of the experiments.

**Figure 5:**
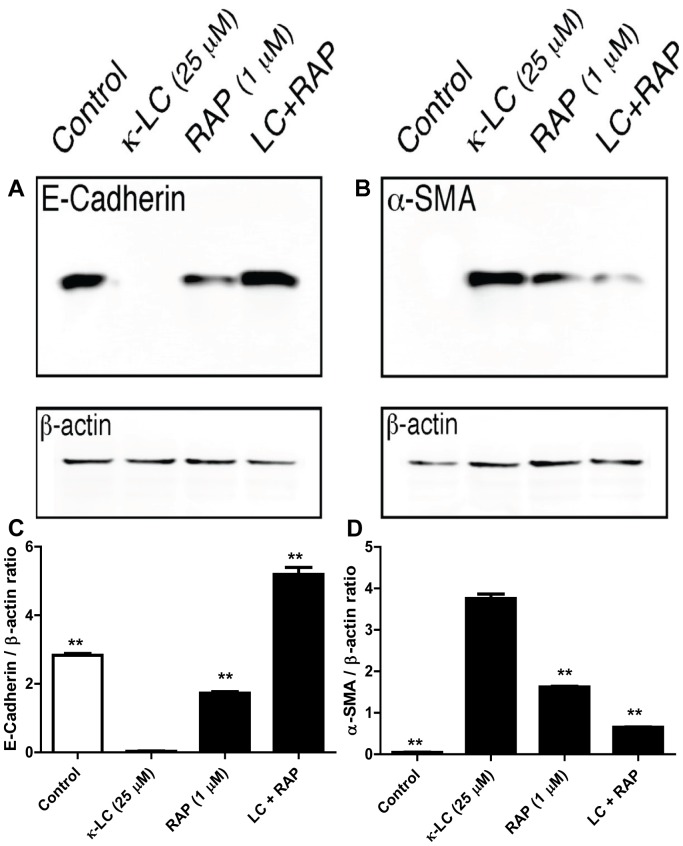
Effect of receptor-associated protein (RAP) on the expression of E-cadherin and α-SMA in myeloma light chain exposed human renal proximal tubule epithelial cells. RAP, 1 µM, prevented LC induced decrease in the epithelial cell marker E-cadherin (**A**), and reduced the increased expression of myofibroblast marker α-SMA (**B**), demonstrating that RAP protected cells from LC-induced EMT (representative blot from 3 experiments). Quantitative densitometric measurement of the effect of RAP pre-treatment followed by κ-LC in HK2 cells on the production of E-cadherin (**C**) and α-SMA (**D**) proteins. **p<0.01 compared to κ-LC alone.

HK-2 cells exposed to 25 µM κ-LC for 48 h exhibited a significant reduction in the expression of the epithelial cell marker E-cadherin, and a significant increase in the mesenchymal marker α-SMA, indicating that another consequence of LC toxicity on PTC is epithelial-to-mesenchymal transformation [9]. Treatment of the cells with human RAP for 6 h prior to LC exposure and subsequent co-treatment achieved preservation of the expression of E-cadherin and prevented the expression of α-SMA (Figure 5). Surprisingly, the treatment of cells with RAP alone seemed to partially suppress the expression of cadherin and partially stimulate the expression of SMA, suggesting the possibility that RAP by itself may initiate partial EMT. However, when combined with LCs, RAP completely restored the expression of E-cadherin, and nearly completely suppressed the expression of α-SMA in LC-exposed cells. This experiment indicates that LC-induced EMT is also dependent on endocytosis, and prevention of endocytosis by adding RAP in the exposure medium prevents EMT.

## Discussion

RAP is a 39-kDa protein that universally inhibits ligand interaction with receptors that belong to the LDL receptor family including megalin [19]. RAP has long been associated with megalin and LDL receptor as a chaperone with a key role in the biosynthesis and intracellular transport of endocytic receptors. Under physiological conditions, it serves as a molecular chaperone/escort protein for these receptors to prevent premature interaction of ligands with the receptors and thereby ensures their proper trafficking through the secretory pathway [24]. It acts as a receptor antagonist and prevents the association of newly synthesized receptors with their ligands during transport to the cell surface. This mechanism seems to be particularly required in cell types that express both the receptor and the ligand to prevent premature receptor-ligand interaction in the secretory pathway that could interfere with proper export of the receptors to the cell surface [25]. It also plays a role in promoting the proper folding of these receptors, a function that maybe unrelated to its ability to inhibit ligand binding [26].

Megalin is a member of the low-density lipoprotein receptor gene family and is abundant in the proximal tubule cell membrane [17]. Like other members of the family, megalin is an endocytic receptor, and along with and sometimes in tandem with cubilin it binds a number of ligands including free light chains isolated and purified from the urine of patients with multiple myeloma [1], [2], [6], [12], [17]. Megalin also binds RAP that serves as an exocytic traffic chaperone and inhibits ligand binding to the receptor [25], [27]. RAP inhibits the binding of virtually all ligands to megalin, including cubilin, although its effect on ligand binding maybe variable. For example, it inhibits the endocytosis of albumin in the proximal tubule cells *in vitro* by ∼40 to 60% [21], [28], while our studies here show ∼88% inhibition of LC endocytosis.

RAP inhibition of megalin ligands may not be universal. For example, RAP at 1 µM in the medium failed to inhibit the endocytosis of insulin, another well-known megalin ligand, in rat kidney PTCs [22]. In another study, RAP at 1 µM in the medium only partially inhibited megalin-dependent internalization of cadmium-metallothionein (by 30.9±6.6%) although this was sufficient to avert cadmium cytotoxicity in cultured renal proximal tubule cells [20]. More recently, inhibition of cadmium-metallothionein endocytosis by RAP in kidney proximal tubule cells was also shown to reduce the toxic effects of cadmium on the kidney in mice *in vivo* [29]. RAP may affect endocytosis in other receptor systems as well, as it was recently shown to inhibit sortilin and mannose-6- receptor mediated endocytosis of alpha-galactosidase in renal endothelial cells *in vitro* [30].

The effect of RAP on LC endocytosis and LC-mediated cytotoxic events has not been evaluated previously. Experiments presented here show that RAP markedly inhibits both the endocytosis and cytotoxicity of LC as expected of most of the ligands of this receptor system, although blocking albumin endocytosis by RAP failed to inhibit albumin-induced TGF-β secretion in PTC [21], a findings that is dissimilar to the LC behavior observed in our studies here. In contrast, the experiments presented here showed that RAP 1 µM reduced the association of total FITC-LC with HK-2 cells nearly completely and reduced acid non-washable (presumed to be endocytosed by cells) by ∼88% (Figure 2.) Thus, in these experiments the effect of RAP on FITC-labeled LC was more than twice as effective than its effect on the uptake of Alexa Fluor 488-coupled metallothionein by cultured renal proximal tubule cells (WKPT-0293 Cl.2 cells) [20]. The marked inhibition of LC endocytosis in the presence of RAP further suggests that in HK-2 cells megalin-dependent endocytosis is the predominant pathway for LC internalization and there is no significant alternate pathway for LC entry in human proximal tubule cells. Our findings here further add to the evidence that LCs are ligands for megalin and cubilin, and are consistent with our previous observation that silencing the megalin and cubilin expression in human kidney cells completely abolished the uptake of free LCs by these cells [23].

We had previously observed that maneuvers that interfere with LC endocytosis protect kidney proximal cells from toxicity [1]–[3], [5], [7], [13], [23]. In these studies we had found that LC endocytosis in PTC can be blocked by rendering the medium hypertonic by sucrose, or after adding to the medium anti-cubilin or anti-megalin antibodies, or the vacuolar H-ATPase inhibitor bafilomycin disrupting vesicular trafficking [1]–[3], [7], [13]. Later, we also showed that silencing megalin and cubilin genes simultaneously by siRNA in cultured PTC nearly abolished the expression of both megalin and cubilin, completely blocked LC endocytosis and prevented LC-induced cytotoxic phenomena including elaboration of inflammatory cytokines, and prevented EMT [9]. Thus, regardless of the nature of the interference, blocking LC endocytosis protected PTC from LC toxicity [3], [5], [7], [13], [23]. Our experiments here demonstrate that blocking LC endocytosis by the addition of RAP in the medium also prevents cytotoxic effects of LC on PTC. These observations confirm and complement our previous observations that LC endocytosis is a prerequisite for its toxic effects in kidney PTCs.

Protein overload as a result of increased endocytosis of filtered proteins in the proximal tubules has been shown to induce cytokine production by renal PTCs, leading to tubulointerstitial injury [5], [31]–[33]. This has been proposed as a major mechanism for the progression of kidney disease in patients with proteinuria [31]–[34]. The kidney disorders in multiple myeloma are often associated with LC proteinuria as a result of “overflow proteinuria”. Excessive LC production by the malignant clone of plasma cells leads to presentation of large amount of LC to the proximal tubule and overloads the endocytic pathways triggering cell stress responses, such as phosphorylation of MAPKs, activation of nuclear transcription factors, induction of inflammatory/proinflammatory cytokines, and EMT [5], [6], [9], [12]. In an animal model, we have demonstrated that LC proteinuria even in modest concentrations may contribute to tubulointerstitial inflammation, the main process responsible for the progression of chronic kidney disease [11]. The cytotoxic phenomena observed in these studies with LC concentrations that can be seen in patients with moderate renal insufficiency but without an overproduction disorder such as myeloma raise the possibility that LC nephrotoxicity may contribute to the progression of chronic kidney disease with modest LC proteinuria, such as diabetes [35]. Our studies with RAP show that regardless of the type of the maneuver used to block endocytosis, the result is protection from cytotoxic phenomena, and suggest that inhibiting excessive endocytosis in the PTC maybe explored as a potential therapeutic strategy. The findings reported by Wolff et al that blocking endocytosis of metallothionein-cadmium complexes by RAP in the medium protects kidney cells from cadmium toxicity is consistent with this idea [20].

Thus, the experiments here show that RAP markedly inhibits the endocytosis of LC in HK-2 cells most probably by inhibiting its binding to its endocytic receptors megalin/cubilin, adding to the evidence that LCs are ligands for this receptor system [1], [2], [23]. Blocking LC endocytosis prevents HK-2 cells from LC-induced stimulation of MAPKs and cytokine production, and protected HK-2 cells from undergoing LC-induced EMT. LC protein overloading may be an important mechanism of proximal tubule responses that contribute to tubulointerstitial disease and protecting PTCs from excessive endocytosis of filtered proteins may be a novel strategy to ameliorate tubulointerstitial inflammation in kidney diseases with proteinuria.
